# Supplementary material for: FAM122A ensures cell cycle interphase progression and checkpoint control by inhibiting B55α/PP2A through helical motifs
Source: Nat Commun. 2024 Jul 10;15:5776. doi: 10.1038/s41467-024-50015-7 (PMC11233601; doi:10.1038/s41467-024-50015-7)
Supplement: Supplementary file 1 — Supplementary Information [file 41467_2024_50015_MOESM1_ESM.pdf]

A Phylogenic tree

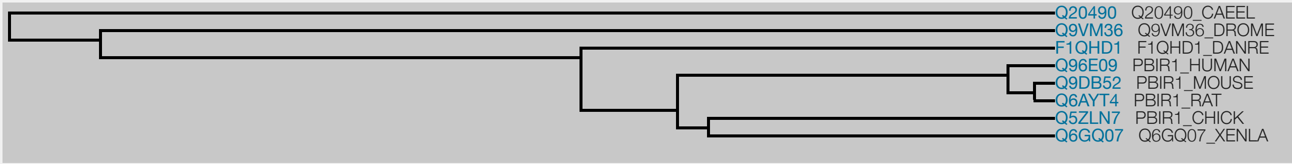

B Alignments

```
Q96E09 PBIR1_HUMAN     1 --------------------------------------------------MAQEKMELDL   10
Q9DB52 PBIR1_MOUSE     1 --------------------------------------------------MAQEKMELDL   10
Q6AYT4 PBIR1_RAT       1 --------------------------------------------------MAQEKMELDL   10
Q5ZLN7 PBIR1_CHICK     1 --------------------------------------------------MAQEKMELDL   10
Q6GQ07 Q6GQ07_XENLA    1 --------------------------------------------------MAQEKMELDL   10
F1QHD1 F1QHD1_DANRE    1 -------------------------------MNN-------------QGIMSQEKMELDL   16
Q9VM36 Q9VM36_DROME   41 ------------LSVPII-RTPPAGKSGKTPMTTRAAAAAALAAKEHEQTPKKSQQVVDI   87
Q20490 Q20490_CAEEL   61 AFRNSPYHAQSPLSAVLFQRPDFLSSEGDRPMSYPSMS------------------SFST  102
                                                                                  ..

Q96E09 PBIR1_HUMAN    11 ELPPGTGGSPAEGGGSG-------GGGGLRRSNSAPLIHGLSDTSPVFQAEAPSARRNST   63
Q9DB52 PBIR1_MOUSE    11 ELPAG--ASPAEGGGPG--------GGGLRRSNSAPLIHGLSDSSPVFQAEAPSARRNST   60
Q6AYT4 PBIR1_RAT      11 ELPAGTGASPAEGGGPG--------SGGLRRSNSAPLIHGLSDSSPVFQAEAPSARRNST   62
Q5ZLN7 PBIR1_CHICK    11 ELPPGSAAAPSD-------------GGGLRRSNSAPLIHGLSDNSQVFQGSVLRTRRNST   57
Q6GQ07 Q6GQ07_XENLA   11 EMPG-----PPS-------------DGNLRRSNSAPLINGLSDNSQVFQTDVIRARRNST   52
F1QHD1 F1QHD1_DANRE   17 DIPTALVQ----------------SDGQLRRSNSEPMINGLSDASQVFQREVLRSRRNST   60
Q9VM36 Q9VM36_DROME   88 QMPNAPSID-----CFG-------KNIHIR---SQPNSR-EVSPAPVFNIFTPRARRYSA  131
Q20490 Q20490_CAEEL  103 NVPDSENSSRSSVDGYNLKDTTSEKIKQFRENNKRKTSNSSSEEEPETEEGDPS-RKLSS  161
                         ::*                         :*   .    .   .     :      *: *:

Q96E09 PBIR1_HUMAN    64 TFPSRHGLLLPASPVRMHSSRLHQIKQEEGMDLI-NRETVHEREVQTAMQISHSWEESFS  122
Q9DB52 PBIR1_MOUSE    61 TFPSRHGLLLPASPVRMHSSRLHQIKQEEGMDLI-NRETVHEREVQTAMQISHSWEESFS  119
Q6AYT4 PBIR1_RAT      63 TFPSRHGLLLPASPVRMHSSRLHQIKQEEGMDLI-NRETVHEREVQTAMQISHSWEESFS  121
Q5ZLN7 PBIR1_CHICK    58 TVMNRHSLFVPSSPIRIPSSRLHQIKQEEGMNLM-NRETVHEREVQVAMQMSQSWEESLS  116
Q6GQ07 Q6GQ07_XENLA   53 TVVNRQSLVVPSSPIRISSSRLHQIKQEEGVDLMINRETAHEREVQVAMQMSQSWEESLN  112
F1QHD1 F1QHD1_DANRE   61 TVVRP--NVVPSSPVRVPSTRLQRIKQEEGVDVM-NRETAHEREVQAAMQMSQSWEESLS  117
Q9VM36 Q9VM36_DROME  132 SYSPLTT--AANGATLCLTPRVSQLRQEECADLN-SREVNHEREVHREIQISQSWEDLTL  188
Q20490 Q20490_CAEEL  162 K-S-AEDK-FVGRGIDAPRGRIANIRRESSCS--VDSEAAHERLTKASQQVSTGFDDIAL  216
                         .                   *: .:::*.  .   . *. ***  .:   *:* .:::

Q96E09 PBIR1_HUMAN   123 LSDNDVEKS---A--------------SPKRIDFIPVSPAPSPTRGI--GK-QCFSPSLQ  162
Q9DB52 PBIR1_MOUSE   120 LSDNDVEKS---A--------------SPKRIDFIPVSPAPSPTRGI--GK-QCFSPSLQ  159
Q6AYT4 PBIR1_RAT     122 LSDNDVEKS---A--------------SPKRIDFIPVSPAPSPTRGI--GK-QCFSPSLQ  161
Q5ZLN7 PBIR1_CHICK   117 LSNNDFEKS---S--------------SPKQVDFVPVSPAPSPTRGI--GK-QCFSPSLQ  156
Q6GQ07 Q6GQ07_XENLA  113 LSDNDLDKS---S--------------SPKRIDFVPVSPAPSPTRGI--GK-QCFSPSLQ  152
F1QHD1 F1QHD1_DANRE  118 LSDNDLEKSSS-S--------------SPKRIDFVPVSPAPSPTRGI--GKKQCFSPSLQ  160
Q9VM36 Q9VM36_DROME  189 VAENWSCKSDEFS--------------NPLQVSLPP----------T--GTTSCSSPSPT  222
Q20490 Q20490_CAEEL  217 DSERSPGTSAEFRRRAPSFNTVIGEPISVVTNAFIPHSCSPSPTRQQADLIKQCYSPSTQ  276
                          ::.   .*                  .     : *                ** ***

Q96E09 PBIR1_HUMAN   163 SFVSSNGLPPSPIPSP------TT---RFTTRRSQSPINCIRPSVLGPLKRKCEMETEYQ  213
Q9DB52 PBIR1_MOUSE   160 SFVSSNGLPPSPIPSP------TT---RFTTRRSQSPINCIRPSVLGPLKRKCEMETDYQ  210
Q6AYT4 PBIR1_RAT     162 SFVSSNGLPPSPIPSP------TT---RFTTRRSQSPINCIRPSVLGPLKRKCEMETDYQ  212
Q5ZLN7 PBIR1_CHICK   157 SLVSSSGLPPSPSPSP------TR---RFSSRRSQSPINCIRPSVLGPIKRKGVTEMEDH  207
Q6GQ07 Q6GQ07_XENLA  153 ILVSSNGLPPSPTPSP------TR---RFATRRSQSPINCIRPSSLGPLKRKGDMEIESQ  203
F1QHD1 F1QHD1_DANRE  161 ILVSSNGLTPSPVPSP------TR---RF--RRSQSPINCIRPGILGTLKRKGEMETESQ  209
Q9VM36 Q9VM36_DROME  223 NNRAGMRLPYSPSPT-------RR---TFATRRSMSPI-AMRPSQLGPVKRKFELDDNPT  271
Q20490 Q20490_CAEEL  277 QMVRPN-ISYSPSPRESPAQSPTRHAHKLKFQRAESPI------CRQPIKRKLTVTTLAE  329
                                :  ** *              :  :*: ***          :***

Q96E09 PBIR1_HUMAN   214 PK------------RFFQGITNMLSSDVAQLSDPGVCVSSD--TLDGNS-----------  248
Q9DB52 PBIR1_MOUSE   211 PK------------RFFQGITNMLSSDVAQLSDPGVCVSSD--TLDGNS-----------  245
Q6AYT4 PBIR1_RAT     213 PK------------RFFQGITNMLSSDVAQLSEPGVCVSSD--TLDGNS-----------  247
Q5ZLN7 PBIR1_CHICK   208 PK------------RLFQGSTNMLSSEALHQPDLGGCLSAH--TLDDNR-----------  242
Q6GQ07 Q6GQ07_XENLA  204 PK------------RLFQGTTNMLSPDVSHLSDLSACLSSE--NLDGSS-----------  238
F1QHD1 F1QHD1_DANRE  210 PK------------RLFQGTTNMLSTEVSHSPELNTCLSPD--LLDGSL-----------  244
Q9VM36 Q9VM36_DROME  272 QGSNWSVYSPPPVKKIFTESRG---SSPVCQSPSSVCPSPDSGTFDGRLTPKLFISKLCT  328
Q20490 Q20490_CAEEL  330 ----------TDIKRLFVPRSNTS---PLVTDKTFPYPQNVGQLFESASTS---------  367
                                       ::*    .                .     ::.

Q96E09 PBIR1_HUMAN   249 -----------SSAGSSCNSPAKVS--TTTDSPVS-----PAQAASPFIPLDELSSK---  287
Q9DB52 PBIR1_MOUSE   246 -----------SSAGSSCNSPAKVS--TTTDSPVS-----PAQAASPFIPVDELSSK---  284
Q6AYT4 PBIR1_RAT     248 -----------SSAGSSCNSPAKVS--TTTDSPVS-----PAQAASPFIPVDELSSK---  286
Q5ZLN7 PBIR1_CHICK   243 -----------SSAGSSCDSPAEAG--ASTGSPVS-----LSDSRSPFLPVDLTAKLPID  284
Q6GQ07 Q6GQ07_XENLA  239 -----------SSLGSSCDSPAKNI--VPESPPSN-----------FFMPVDRHSPK---  271
F1QHD1 F1QHD1_DANRE  245 -----------SSVGSSSDSPAKME--GVSPS----------PSNSPLTPLQDLSPK---  278
Q9VM36 Q9VM36_DROME  329 SNSVSGNGINNNSSSSGCPSPVSASPGGVGSGPNLEAAMCLVSGGSQSQSIDEGISIPES +118  388
Q20490 Q20490_CAEEL  368 ------------SEFSSFSVPH--SPG-----PL--RAMTPLSSVSVGD-QEENSPSVKD +48   405
                                     *  *.   *                              :
```

Supp. Fig. 1

Suppl. Fig. 1. FAM122A is first found in *Bilateria* animals, and the SLiM is Fully conserved. Phylogenetic tree (**A)** and sequence alignment **(B)** were generated using the UniProt sequence alignment toolkit. An "*" (asterisk) indicates positions that have a single, fully conserved residue. A ":" (colon) indicates conservation between groups of strongly similar properties - scoring > 0.5 in the Gonnet PAM 250 matrix. A "." (period) indicates conservation between groups of weakly similar properties - scoring =< 0.5 in the Gonnet PAM 250 matrix.

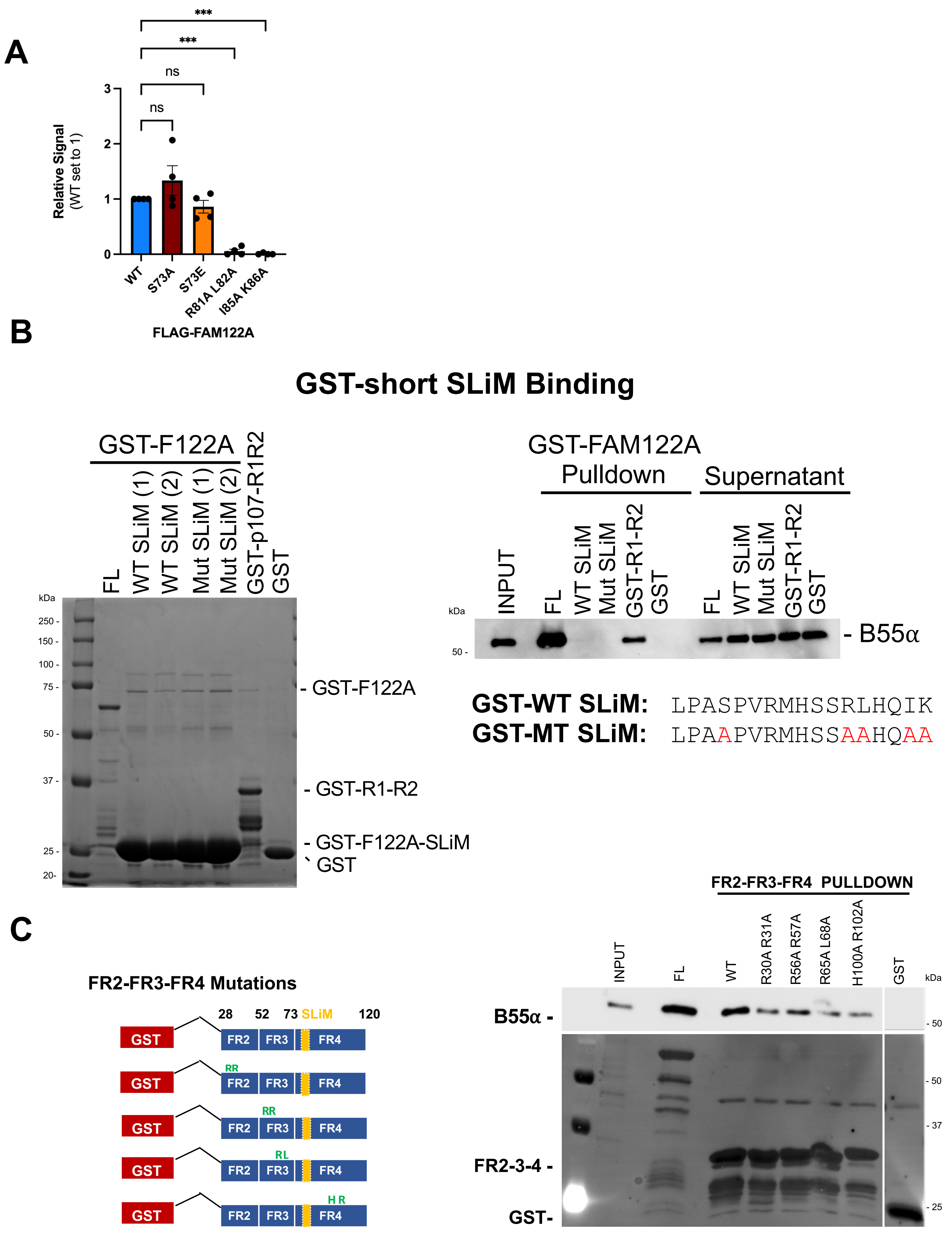
A
***
***
ns
ns
Relative Signal
(WT set to 1)
0
1
2
3
WT
S73A
S73E
R81A L82A
I85A K86A
FLAG-FAM122A
B
GST-short SLiM Binding
GST-F122A
FL
WT SLiM (1)
WT SLiM (2)
Mut SLiM (1)
Mut SLiM (2)
GST-p107-R1R2
GST
kDa
250 -
150 -
100 -
75 -
50 -
37 -
25 -
20-
- GST-F122A
- GST-R1-R2
- GST-F122A-SLiM
` GST
GST-FAM122A
Pulldown
Supernatant
INPUT
FL
WT SLiM
Mut SLiM
GST-R1-R2
GST
FL
WT SLiM
Mut SLiM
GST-R1-R2
GST
kDa
50 -
- B55α
GST-WT SLiM: LPASPVRMHSSRLHQIK
GST-MT SLiM: LPAAPVRMHSSAAHQAA
C
FR2-FR3-FR4 Mutations
28
52
73
SLiM
120
GST
FR2
FR3
FR4
RR
RR
RL
H R
FR2-FR3-FR4 PULLDOWN
INPUT
FL
WT
R30A R31A
R56A R57A
R65A L68A
H100A R102A
GST
kDa
B55α -
- 50
- 50
- 37
FR2-3-4 -
GST-
- 25

Supp. Fig. 2

Suppl. Fig. 2. A. While the SLiM is essential for binding B55α, sequences beyond FR4 contribute contacts. A. Quantitation of relative Myc-B55α binding to FLAG-FAM122A WT and MTs in Myc-IPs of HEK293T cells cotransfected with indicated constructs (n=4) and analyzed for statistical significance with a One-way ANOVA (two-sided) with a Dunnett's test for multiple comparisons. WT:S73A p-value = 0.2510 (ns). WT:S73E p-value = 0.8647 (ns). WT:R81A L82A p-value = 0.0005. WT:I85A K86A p-value = 0.0003 **B.** (Left) A Coomassie blue-stained gel of full-length GST-tagged FAM122A, GST-p107 R1R2, GST, and 17 residue peptides containing the FAM122A SLiM (WT and MT) fused to GST. (Right) GST-Pulldowns using the indicated GST-construct demonstrate that the SLiM alone is insufficient for binding B55α. **C.** GST-FAM122A pulldown assays with the indicated variant constructs eliminating positively charged amino acid pairs. Source data are provided as a Source Data file.

A AlphaFold2_Advanced 4 top models

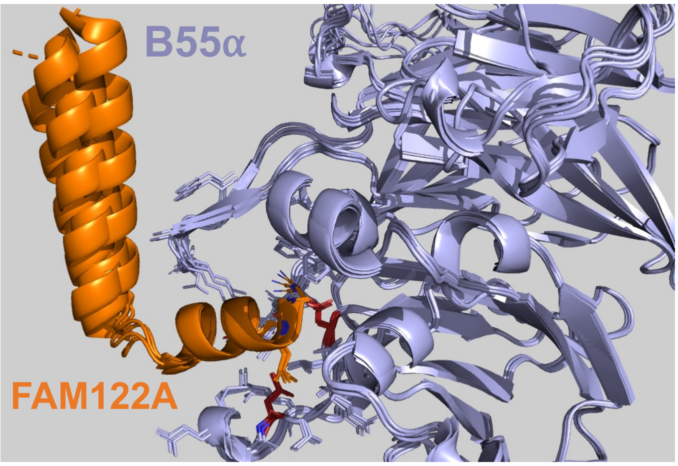

B

AlphaFold2 RL**HQ**I WT

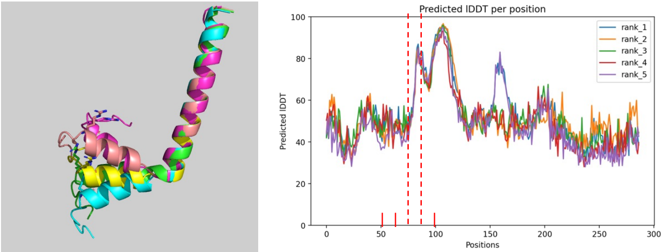

AlphaFold2 RL**AA**I MT

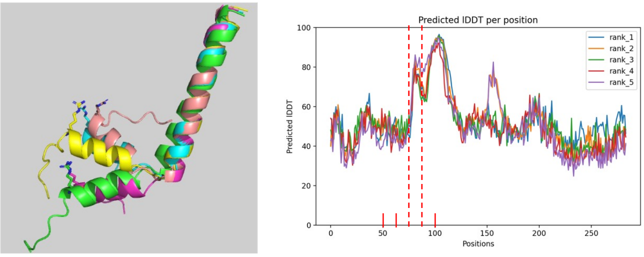

AlphaFold2 RL**PP**I
α-helix MT breakers

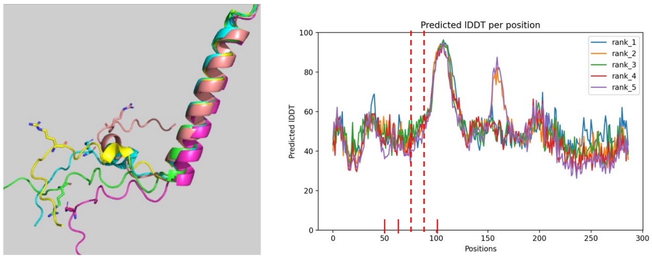

AlphaFold2 RL**GG**I
α-helix MT breakers

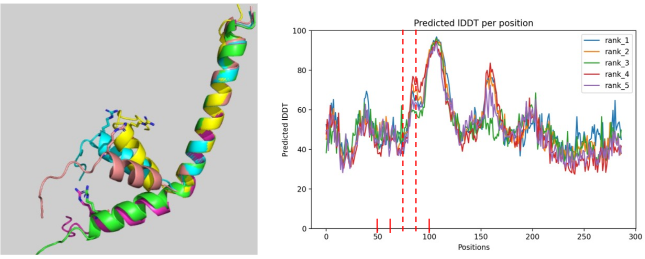

Suppl. Fig. 3

Suppl. Fig. 3*. AlphaFold2_advanced* top models and the prediction of the variable SLiM residue mutations in FAM122A . A. *AlphaFold2_advanced* prediction models (top 4) of FAM122A binding to B55α **B.** ColabFold (*AlphaFold2_advanced*) prediction models and scores for FAM122A WT and mutations to disrupt helix formation. The five models are superimposed in each mutant. The region of helix-1 region is flanked by red-dashed lines, demonstrating lower structural confidence scores.

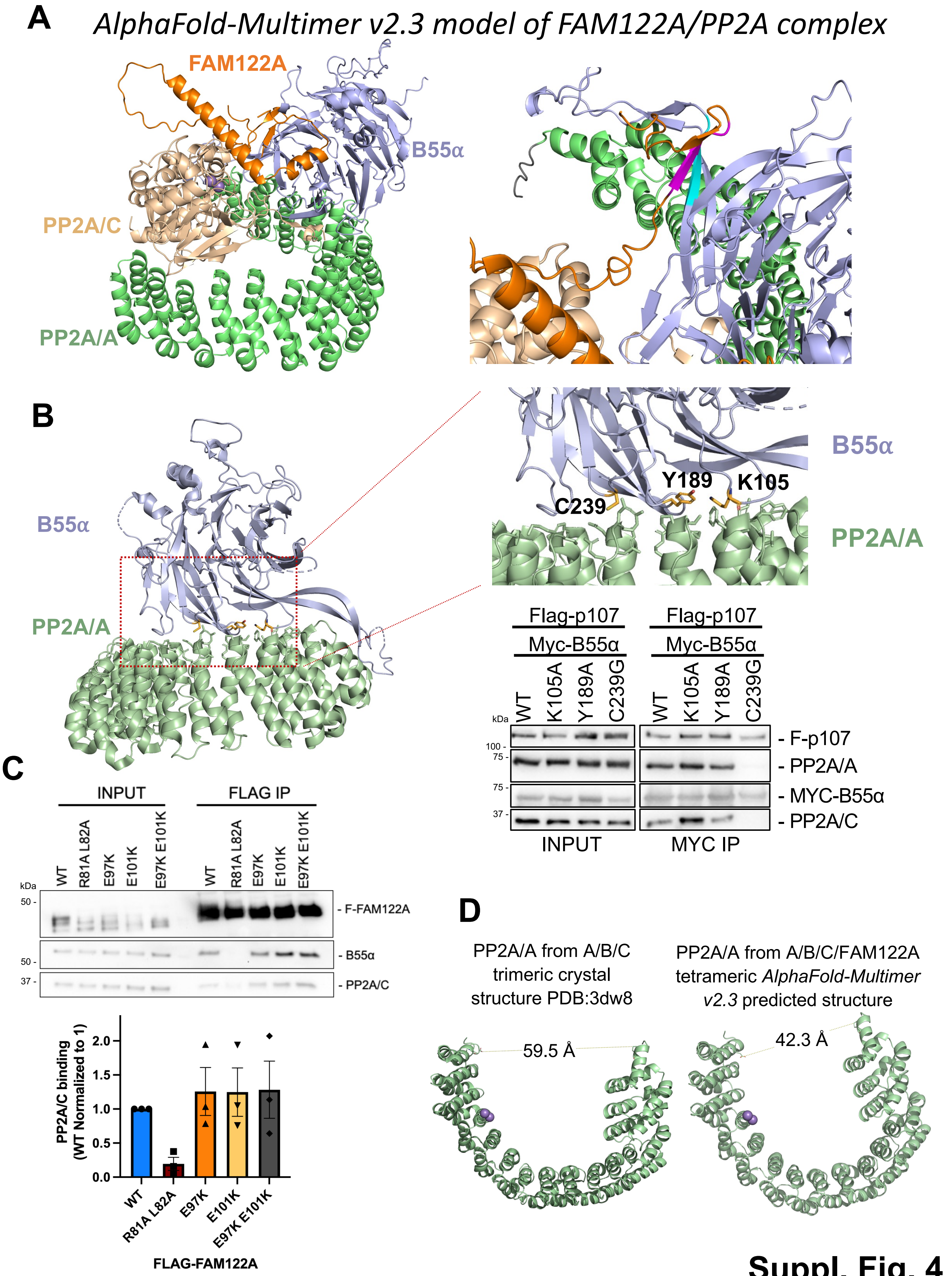
A
AlphaFold-Multimer v2.3 model of FAM122A/PP2A complex
FAM122A
B55α
PP2A/C
PP2A/A
B
B55α
PP2A/A
Y189
K105
C239
B55α
PP2A/A
Flag-p107
Myc-B55α
Flag-p107
Myc-B55α
WT
K105A
Y189A
C239G
WT
K105A
Y189A
C239G
kDa
100 -
75 -
75 -
37 -
- F-p107
- PP2A/A
- MYC-B55α
- PP2A/C
INPUT
MYC IP
C
INPUT
FLAG IP
WT
R81A L82A
E97K
E101K
E97K E101K
WT
R81A L82A
E97K
E101K
E97K E101K
kDa
50 -
50 -
37 -
- F-FAM122A
- B55α
- PP2A/C
PP2A/C binding (WT Normalized to 1)
2.0
1.5
1.0
0.5
0.0
WT
R81A L82A
E97K
E101K
E97K E101K
FLAG-FAM122A
D
PP2A/A from A/B/C trimeric crystal structure PDB:3dw8
PP2A/A from A/B/C/FAM122A tetrameric AlphaFold-Multimer v2.3 predicted structure
59.5 Å
42.3 Å

Suppl. Fig. 4

Suppl. Fig. 4. Additional views of the PP2A/B55α:FAM122A AlphaFold2 v2.3 model, the development of 'monomeric' B55α mutants, mutation of FAM122A helix-2 glutamic acids, and PP2A/A curvature differences between the tetrameric AlphaFold2 model and the trimeric 3dw8 crystal structure. A. AlphaFold-Multimer v2.3 cartoon model of the FAM122A/PP2A holoenzyme. Additional contacts with the holoenzyme may come from a potential beta-strand in FAM122A with PP2A/A. **B.** The interface of B55α and PP2A/A from the 3dw8 crystal structure used to generate B55α mutants that would render it monomeric. Myc-IPs of Myc-B55α mutants and FLAG-p107 demonstrate the ability of the C239G mutant to bind substrates (p107) while failing to bind the scaffold and catalytic subunits. **C.** FLAG-IPs of indicated FLAG-FAM122A mutants transfected in HEK293T cells to assess the binding of these mutants to B55α and PP2A/C (n=3). Source data are provided as a Source Data file. **D.** Comparison of the curvature of the PP2A/A (scaffold) subunit from the 3dw8 (59.5 Å) crystal structure to the FAM122A/PP2A/B55α tetrameric complex (42.3 Å).

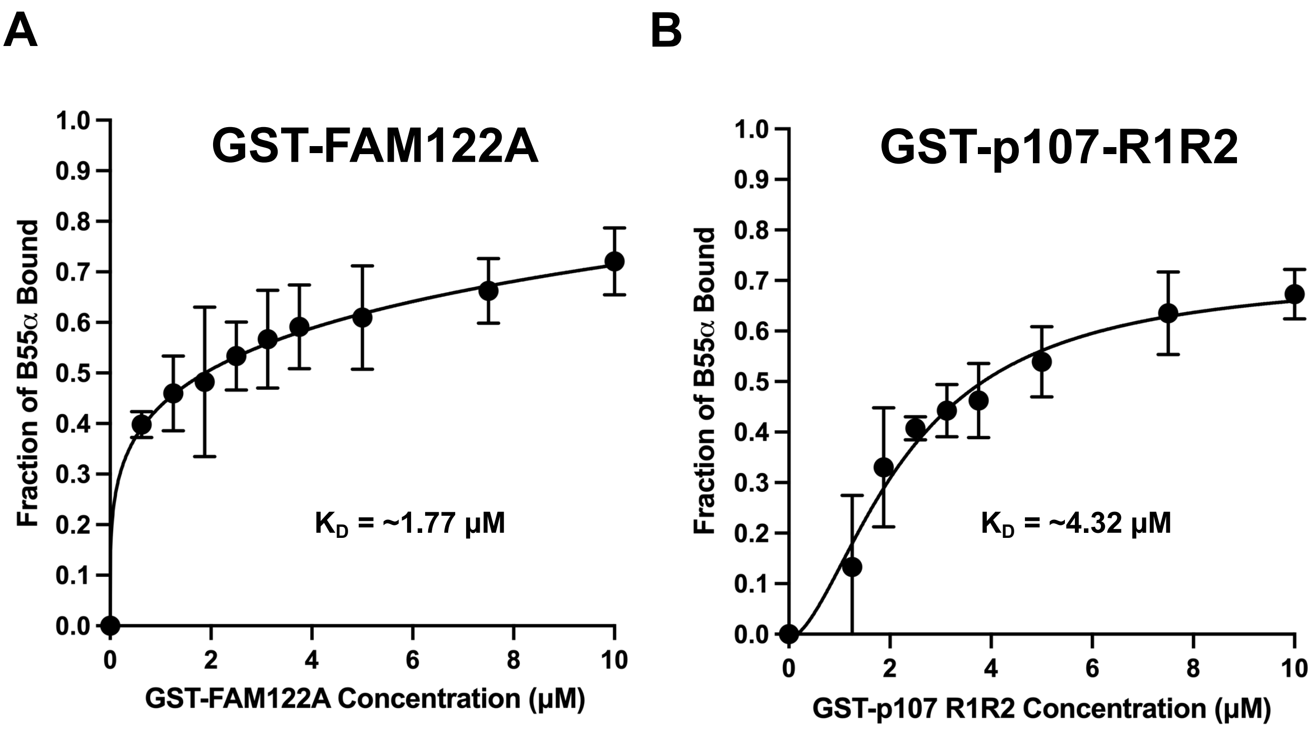
A
GST-FAM122A
Fraction of B55α Bound
K_D = ~1.77 µM
GST-FAM122A Concentration (µM)
B
GST-p107-R1R2
Fraction of B55α Bound
K_D = ~4.32 µM
GST-p107 R1R2 Concentration (µM)

Suppl. Fig. 5

Suppl. Figure 5 Comparison of GST-FAM122A/GST-p107 R1R2: B55α Affinities. A. Estimated affinity of GST-FAM122A to B55α as determined by depletion of B55α from HEK-293T lysate. GST-FAM122A pulldowns were performed overnight at 4°C at the indicated concentrations in a 100 μl final volume with 100 μg of HEK293T lysate. The supernatants were collected and run in SDS-PAGE gels, transferred to PVDF membranes, and probed for B55α to measure depletion and GAPDH as a loading correction. K_D= ~1.77 μM (n=4). **B.** Same experimental design but performed using GST-p107 R1R2. K_D= ~4.32 μM (n=3). Source data are provided as a Source Data file.

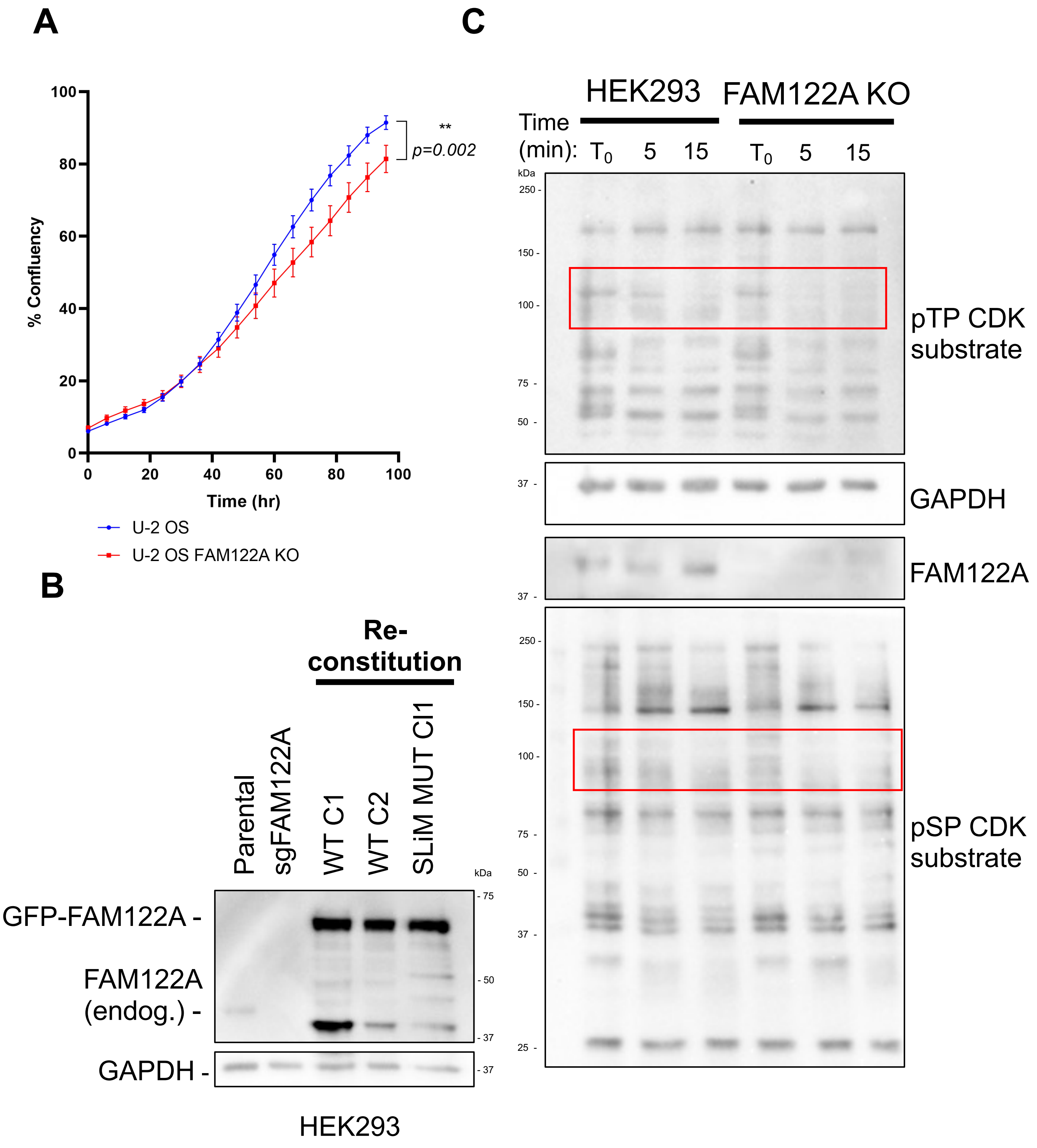
A
100
80
60
40
20
0
% Confluency
0
20
40
60
80
100
Time (hr)
**
p=0.002
U-2 OS
U-2 OS FAM122A KO
B
Re-constitution
Parental
sgFAM122A
WT C1
WT C2
SLiM MUT Cl1
kDa
- 75
- 50
- 37
- 37
GFP-FAM122A -
FAM122A (endog.) -
GAPDH -
HEK293
C
HEK293
FAM122A KO
Time (min):
T_0
5
15
T_0
5
15
kDa
250 -
150 -
100 -
75 -
50 -
37 -
37 -
250 -
150 -
100 -
75 -
50 -
37 -
25 -
pTP CDK substrate
GAPDH
FAM122A
pSP CDK substrate

Supp. Fig. 6

Suppl. Fig. 6. FAM122A Knockout in U2-OS cells, HEK293 FAM122A reconstitution and substrates phosphorylated on CDK SP/TP in protein lysates lacking FAM122A are dephosphorylated with faster kinetics. A. Proliferation curves of FAM122A KO in U-2 OS cells display similar proliferative defects. Statistical significance was addressed using a Wilcoxon matched-pairs signed rank test of the last 10 measurements as in Figure 6. **B.** Western blot for FAM122A in HEK293 cells, KO and WT/MT reconstitution. **C.** Western blot for phospho-CDK substrates (TP and SP) to demonstrate phosphatase activity in FAM122A expressing versus KO cells. Cells were lysed in the absence of phosphatase inhibitors, with inhibitors being added at indicated timepoints. Bands corresponding to pTP and pSP substrates with faster kinetics in FAM122A-KO cells are indicated. Source data are provided as a Source Data file.

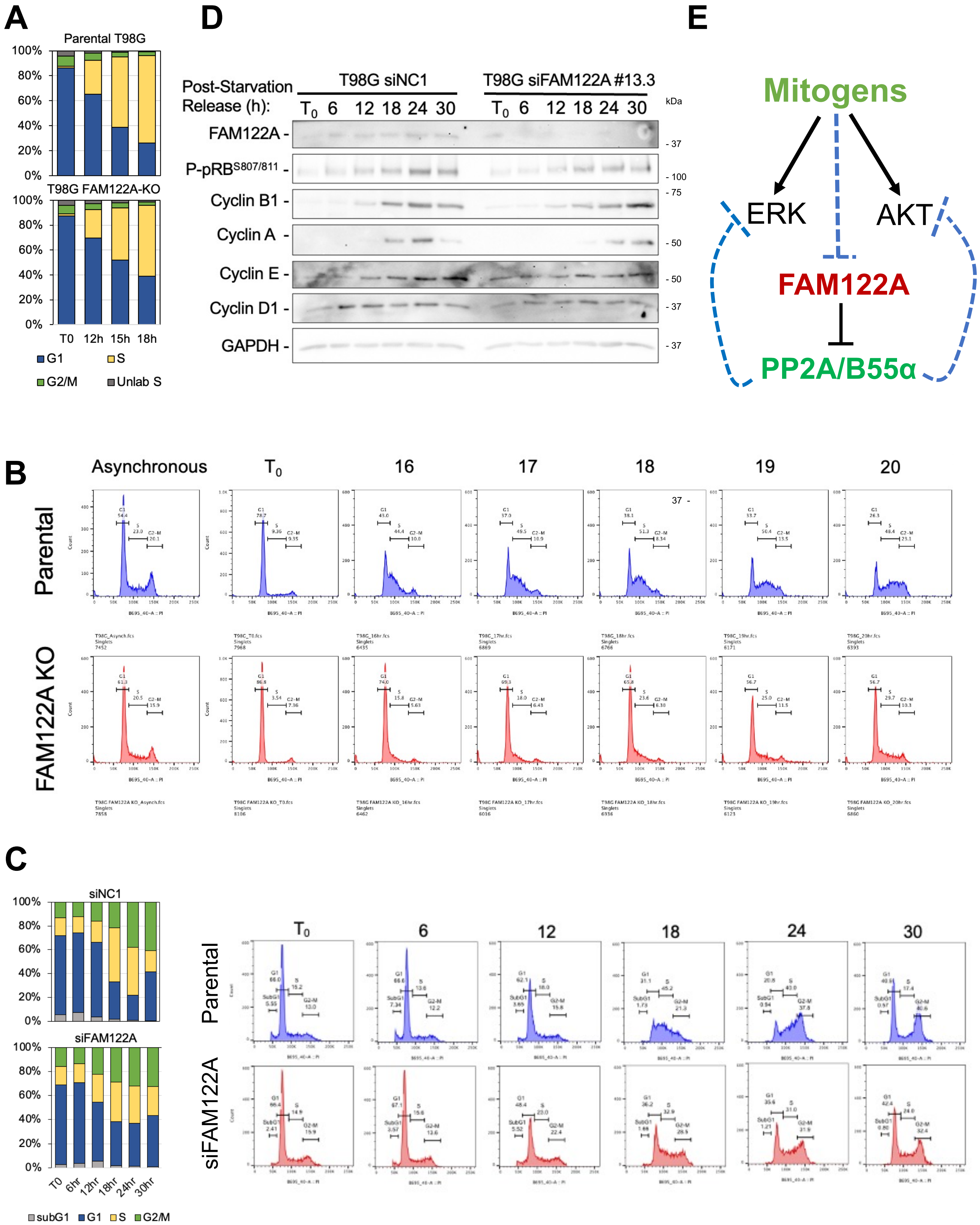
A
Parental T98G
T98G FAM122A-KO
100%
80%
60%
40%
20%
0%
T0 12h 15h 18h
G1
S
G2/M
Unlab S
D
Post-Starvation Release (h):
T98G siNC1
T98G siFAM122A #13.3
T0 6 12 18 24 30
kDa
FAM122A -
P-pRB S807/811 -
Cyclin B1 -
Cyclin A -
Cyclin E -
Cyclin D1 -
GAPDH -
- 37
- 100
- 75
- 50
- 50
- 37
- 37
E
Mitogens
ERK
AKT
FAM122A
PP2A/B55α
B
Asynchronous
T0
16
17
18
19
20
Parental
FAM122A KO
C
siNC1
siFAM122A
T0 6hr 12hr 18hr 24hr 30hr
subG1
G1
S
G2/M
T0
6
12
18
24
30
Parental
siFAM122A

Suppl. Fig. 7. Raw propidium iodide flow cytometry data and knockdown of FAM122A with siRNA in serum-starved T98G cells delays progression through G1/S, pRB phosphorylation, and expression of cyclins. A. Quantitation of cell cycle profiles in the form of bar graphs for PI/EdU flow cytometry data presented in Fig. 7A. **B.** Propidium iodide flow cytometry plots for time points presented in Figure 7B. **C.** (Left) Bar graphs representing % cell cycle phase of time points shown. (Right) PI cell cycle analysis of FAM122A knockdown in T98G cells shows delayed cell cycle progression. **D.** Western blot analysis detailing delays in pRB phosphorylation and expression of cyclins following FAM122A knockdown. Source data are provided as a Source Data file. **E.** Pathway schematic demonstrating the role of FAM122A on B55α attenuation of ERK and AKT activity.

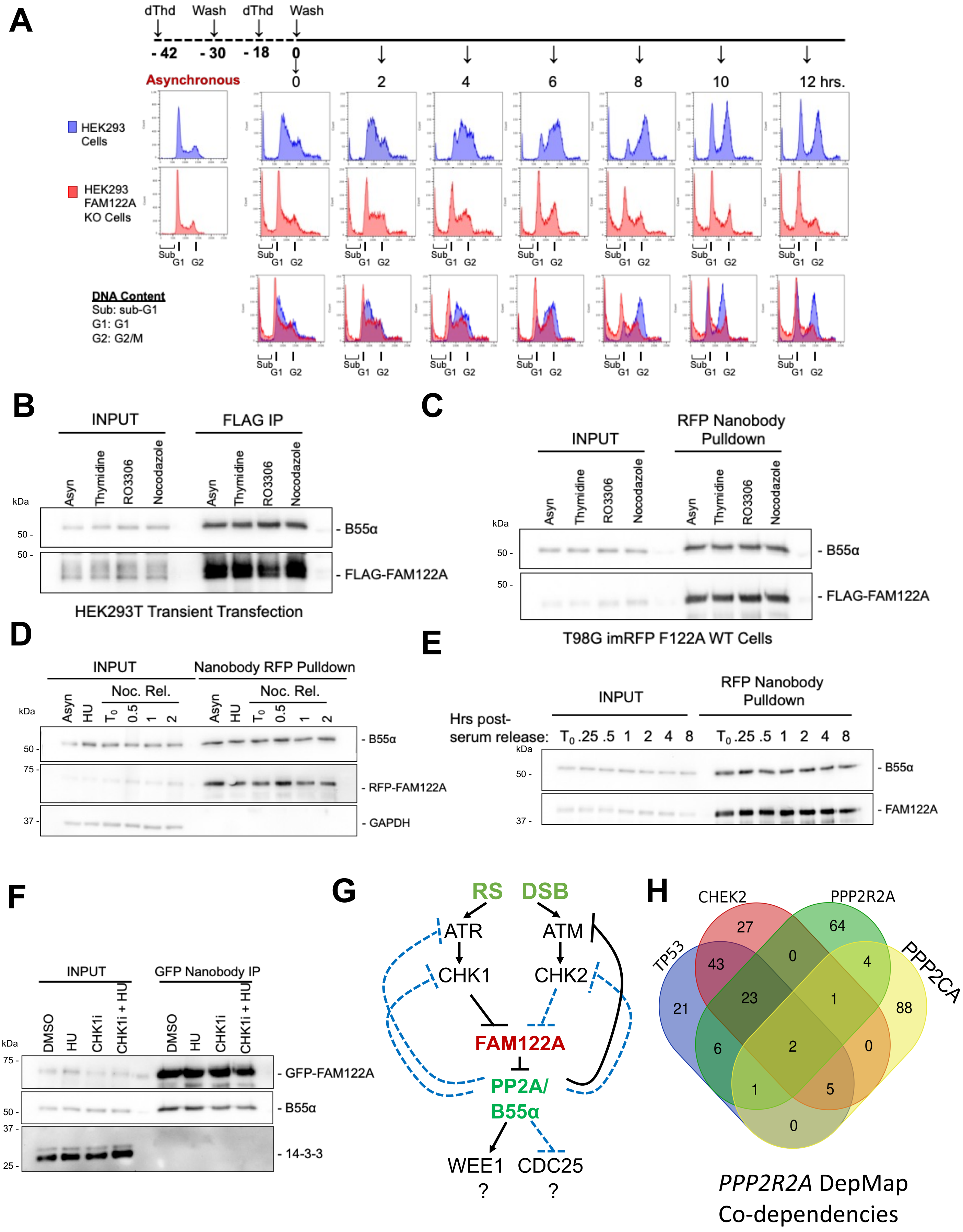
A
dThd Wash dThd Wash
- 42 - 30 - 18 0 2 4 6 8 10 12 hrs.
Asynchronous
HEK293 Cells
HEK293 FAM122A KO Cells
Sub G1 G2
DNA Content
Sub: sub-G1
G1: G1
G2: G2/M
B
INPUT FLAG IP
Asyn Thymidine RO3306 Nocodazole
kDa
50 -
- B55α
- FLAG-FAM122A
HEK293T Transient Transfection
C
INPUT RFP Nanobody Pulldown
- B55α
- FLAG-FAM122A
T98G imRFP F122A WT Cells
D
INPUT Nanobody RFP Pulldown
Noc. Rel.
Asyn HU T0 0.5 1 2
- B55α
- RFP-FAM122A
- GAPDH
E
INPUT RFP Nanobody Pulldown
Hrs post-serum release: T0 .25 .5 1 2 4 8
- B55α
- FAM122A
F
INPUT GFP Nanobody IP
DMSO HU CHK1i CHK1i + HU
- GFP-FAM122A
- B55α
- 14-3-3
G
RS DSB
ATR ATM
CHK1 CHK2
FAM122A
PP2A/ B55α
WEE1 ? CDC25 ?
H
CHEK2 PPP2R2A TP53 PPP2CA
27 64 43 0 4 21 23 1 88 6 2 0 1 5 0
PPP2R2A DepMap Co-dependencies

Supp. Fig. 8

I

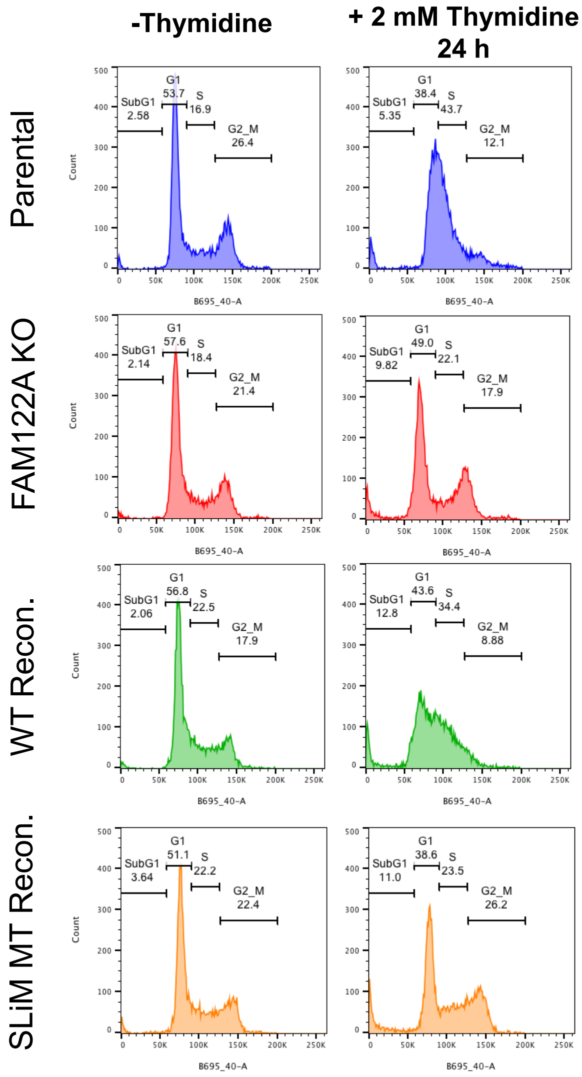

-Thymidine
+ 2 mM Thymidine
24 h
Parental
FAM122A KO
WT Recon.
SLiM MT Recon.
SubG1
2.58
G1
53.7
S
16.9
G2_M
26.4
SubG1
5.35
G1
38.4
S
43.7
G2_M
12.1
SubG1
2.14
G1
57.6
S
18.4
G2_M
21.4
SubG1
9.82
G1
49.0
S
22.1
G2_M
17.9
SubG1
2.06
G1
56.8
S
22.5
G2_M
17.9
SubG1
12.8
G1
43.6
S
34.4
G2_M
8.88
SubG1
3.64
G1
51.1
S
22.2
G2_M
22.4
SubG1
11.0
G1
38.6
S
23.5
G2_M
26.2
Count
B695_40-A

Supp. Fig. 8

Suppl. Fig. 8. Double thymidine block release of FAM122A KO cells and FAM122A is constitutively bound to B55α during the cell cycle and the G1/S and intra-S checkpoints. **A.** Schematic and cell cycle PI profiles of HEK293 and FAM122A KO cells released from a double thymidine block. FAM122A KO cells fail to synchronize like parental HEK293 cells and exhibit a massive increase in SubG1 DNA content. **B-C.** Western blot of pulldowns of FLAG-FAM122A HEK293T and reconstituted RFP-FAM122A T98G cells treated for 24 h with DMSO, 2 mM Thymidine, 10 μM RO3306, or 10 nM nocodazole and probed for B55α binding. **D.** Western blot of RFP-nanbody pulldown of RFP-FAM122A T98G cells treated for 24 h with DMSO, 2mM HU, or 10 nM nocodazole. Nocodazole-treated cells were also released at given time points and probed for B55α binding. **E.** Western blot of RFP-nanobody pulldown of reconstituted RFP-FAM122A T98G cells serum-starved for 72 h at indicated points of serum restimulation and probed for B55α binding. **F.** GFP-FAM122A IP demonstrating absence of global B55α binding regulation in response to replication stress. All blot source data are provided as a Source Data file.**G.** a pathway schematic of FAM122A and B55α that involves regulators of the replication stress response. **H.** Venn diagram showing common hits among DepMap co-dependencies for *PPP2R2A* (B55α), *CHEK2, TP53, PPP2CA*. **I.** Cell cycle PI profiles of thymidine challenged Parental, FAM122A KO, WT/MT reconstitution HEK293 cells, demonstrating a SLiM-dependent rescue of the G1/S arrest (n=3).

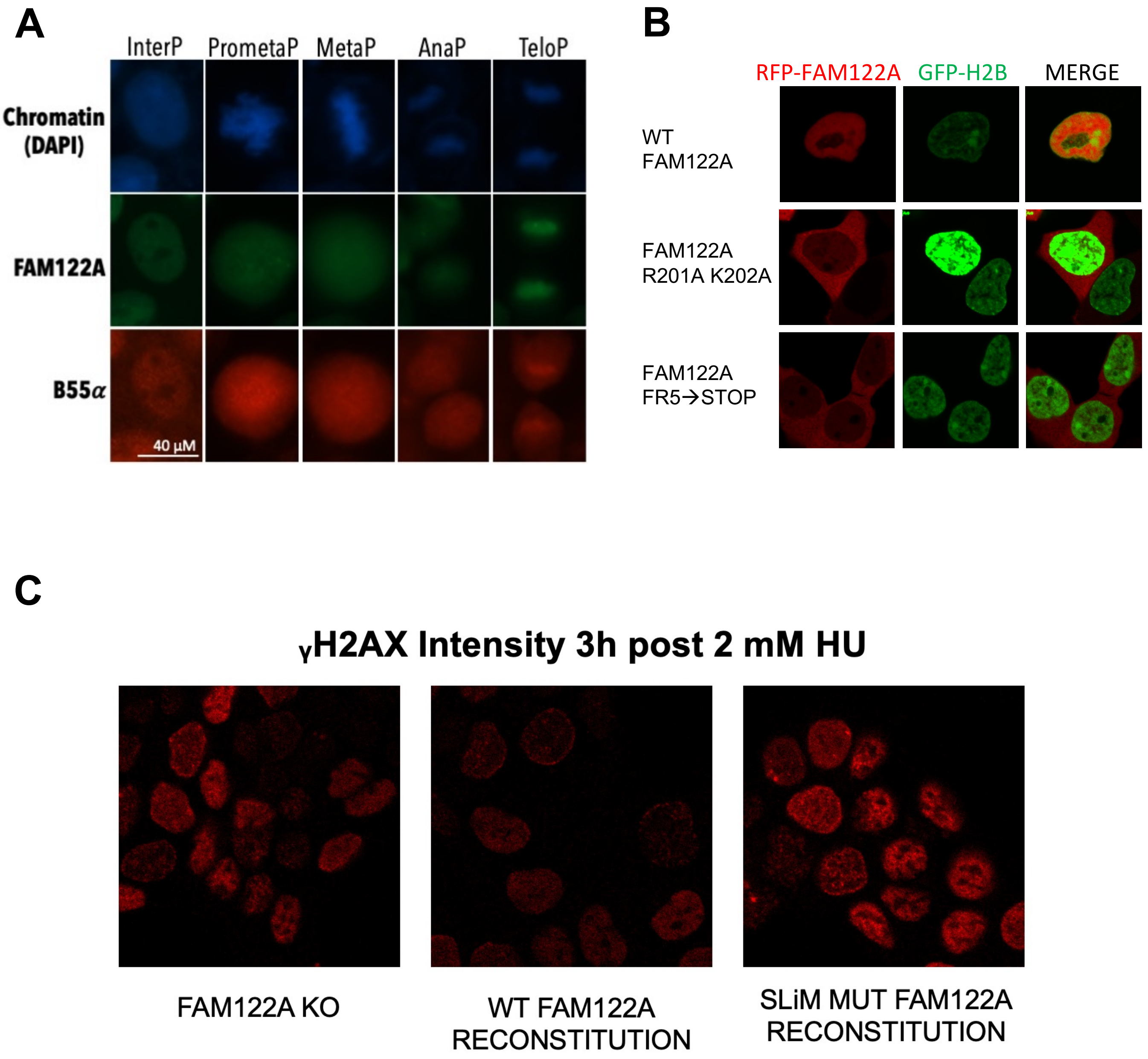
A
InterP
PrometaP
MetaP
AnaP
TeloP
Chromatin (DAPI)
FAM122A
B55α
40 μM
B
RFP-FAM122A
GFP-H2B
MERGE
WT FAM122A
FAM122A R201A K202A
FAM122A FR5→STOP
C
γH2AX Intensity 3h post 2 mM HU
FAM122A KO
WT FAM122A RECONSTITUTION
SLiM MUT FAM122A RECONSTITUTION

Supp. Fig. 9

Suppl. Fig. 9. FAM122A is a nuclear protein in interphase that colocalizes with B55α. A. Immunofluorescence in U-2 OS cells shows that FAM122A and B55α colocalize in the nucleus in interphase. Coinciding with chromosome condensation, no colocalization of FAM122A or B55α with chromatin is observed in prometaphase or metaphase. However, colocalization with chromatin is observed starting in late in anaphase. Chromatin was visualized with DAPI, whereas FAM122A and B55α were visualized using antibodies. **B.** mRFP-FAM122A WT and NLS deficient MT constructs were co-transfected with GFP-h-H2B to determine the dependency of FAM122A on the proposed NLS in HEK293 cells. (63x oil immersion confocal images, 5x zoom factor). **C.** Representative images of γH2AX nuclear intensity in HEK293 FAM122A KO and FAM122A WT and SLiM MUT reconstituted cells corresponding to the quantitation shown in Fig. 9E.
