# Supplementary material for: FAM122A ensures cell cycle interphase progression and checkpoint control by inhibiting B55α/PP2A through helical motifs
Source: Nat Commun. 2024 Jul 10;15:5776. doi: 10.1038/s41467-024-50015-7 (PMC11233601; doi:10.1038/s41467-024-50015-7)
Supplement: Supplementary file 3 — Description of Additional Supplementary Files [file 41467_2024_50015_MOESM3_ESM.pdf]

Description of Additional Supplementary Files

File Name: Supplementary Data 1
Description: Common hits among the ScanProsite search and proteins in the datasets indicated in Fig. 1B.

File Name: Supplementary Data 2
Description: Phosphoproteomics results from induction of B55a expression in HEK293 cells reported in Fig. 1B.

File Name: Supplementary Data 3
Description: Mass spec data corresponding to monomeric B55a vs. PP2A/B55a Holoenzyme pulldowns plotted in Fig. 4C.

File Name: Supplementary Data 4
Description: PyMOL file of Model B55/PP2A Holoenzyme bound to FAM122A

File Name: Supplementary Data 5
Description: Model Coordinates of Model B55/PP2A Holoenzyme bound to FAM122A

File Name: Supplementary Data 6
Description: Key Resource Table
