# Supplementary material for: FAM122A ensures cell cycle interphase progression and checkpoint control by inhibiting B55α/PP2A through helical motifs
Source: Nat Commun. 2024 Jul 10;15:5776. doi: 10.1038/s41467-024-50015-7 (PMC11233601; doi:10.1038/s41467-024-50015-7)
Supplement: Supplementary file 10 — Reporting Summary [file 41467_2024_50015_MOESM10_ESM.pdf]

nature portfolio

Corresponding author(s): Xavier Graña

Last updated by author(s): Jun 4, 2024

Reporting Summary

Nature Portfolio wishes to improve the reproducibility of the work that we publish. This form provides structure for consistency and transparency in reporting. For further information on Nature Portfolio policies, see our Editorial Policies and the Editorial Policy Checklist.

Statistics

For all statistical analyses, confirm that the following items are present in the figure legend, table legend, main text, or Methods section.

n/a	Confirmed	
☐	☒	The exact sample size (*n*) for each experimental group/condition, given as a discrete number and unit of measurement
☐	☒	A statement on whether measurements were taken from distinct samples or whether the same sample was measured repeatedly
☐	☒	The statistical test(s) used AND whether they are one- or two-sided<br>*Only common tests should be described solely by name; describe more complex techniques in the Methods section.*
☒	☐	A description of all covariates tested
☐	☒	A description of any assumptions or corrections, such as tests of normality and adjustment for multiple comparisons
☐	☒	A full description of the statistical parameters including central tendency (e.g. means) or other basic estimates (e.g. regression coefficient) AND variation (e.g. standard deviation) or associated estimates of uncertainty (e.g. confidence intervals)
☐	☒	For null hypothesis testing, the test statistic (e.g. *F*, *t*, *r*) with confidence intervals, effect sizes, degrees of freedom and *P* value noted<br>*Give P values as exact values whenever suitable.*
☒	☐	For Bayesian analysis, information on the choice of priors and Markov chain Monte Carlo settings
☒	☐	For hierarchical and complex designs, identification of the appropriate level for tests and full reporting of outcomes
☒	☐	Estimates of effect sizes (e.g. Cohen's *d*, Pearson's *r*), indicating how they were calculated

Our web collection on statistics for biologists contains articles on many of the points above.

Software and code

Policy information about availability of computer code

Data collection	Protein identification was performed by searching MS/MS data against the Swiss-prot human protein database using andromeda 1.5.6.0 built in MaxQuant 1.6.1.0.<br><br>Raw data were searched using COMET (release version 2014.01) in high resolution mode against a target-decoy (reversed) version of the human proteome sequence database (UniProt; downloaded 2/2020, 40704 entries of forward and reverse protein sequences).<br><br>Some calculations were performed using the Jupyter notebooks provided by ColabFold, which requires inputting the query sequence and choosing options for running the calculation using Google Colab GPUs. We used both the original AlphaFold2 notebook and the AlphaFold2_advanced notebook (https://colab.research.google.com/github/sokrypton/ColabFold/blob/main/beta/AlphaFold2_advanced.ipynb). The latter was developed to model complexes of proteins before the availability of AlphaFold-Multimer. The notebook https://colab.research.google.com/github/sokrypton/ColabFold/blob/main/AlphaFold2.ipynb implements both the original AlphaFold2 trained on single-chain proteins and AlphaFold-Multimer for complexes. We ran AlphaFold2 without templates on the ColabFold notebooks.<br><br>For calculations with AlphaFold-Multimer 2.3 (December 2022), we downloaded code from the DeepMind github repository (https://github.com/deepmind/alphafold) for computations on a Linux workstation with a 24 Gbyte GPU.<br><br>Structures were optimized with AMBER (default in AlphaFold2) and visualized with PyMOL.

Data analysis	Quantification of LC-MS/MS spectra was performed using MassChroQ and the iBAQ method.<br><br>Graphs were generated in Prism Graphpad 9/10 and Excel.<br><br>Densitometric analysis of western blots was performed in Fiji (ImageJ).<br><br>AlphaFold models were analyzed with Pymol.

For manuscripts utilizing custom algorithms or software that are central to the research but not yet described in published literature, software must be made available to editors and reviewers. We strongly encourage code deposition in a community repository (e.g. GitHub). See the Nature Portfolio guidelines for submitting code & software for further information.

Data

Policy information about availability of data

All manuscripts must include a data availability statement. This statement should provide the following information, where applicable:

- Accession codes, unique identifiers, or web links for publicly available datasets
- A description of any restrictions on data availability
- For clinical datasets or third party data, please ensure that the statement adheres to our policy

Data publicly available in a repository:
• The AlphaFold-Multimer model of the tetrameric complex and a PyMOL session are available at https://doi.org/10.5281/zenodo.7739038.
• Mass spectrometry data and associated metadata suitable for specialized repositories has been deposited and will be made publicly available indefinitely through ProteomeXchange.

Data available with the paper or supplementary information:
• All data supporting the findings of this study are available within the paper and its Supplementary Information.

Research involving human participants, their data, or biological material

Policy information about studies with human participants or human data. See also policy information about sex, gender (identity/presentation), and sexual orientation and race, ethnicity and racism.

Reporting on sex and gender	N/A
Reporting on race, ethnicity, or other socially relevant groupings	N
Population characteristics	*Describe the covariate-relevant population characteristics of the human research participants (e.g. age, genotypic information, past and current diagnosis and treatment categories). If you filled out the behavioural & social sciences study design questions and have nothing to add here, write "See above."*
Recruitment	*Describe how participants were recruited. Outline any potential self-selection bias or other biases that may be present and how these are likely to impact results.*
Ethics oversight	*Identify the organization(s) that approved the study protocol.*

Note that full information on the approval of the study protocol must also be provided in the manuscript.

Field-specific reporting

Please select the one below that is the best fit for your research. If you are not sure, read the appropriate sections before making your selection.

☒ Life sciences ☐ Behavioural & social sciences ☐ Ecological, evolutionary & environmental sciences

For a reference copy of the document with all sections, see nature.com/documents/nr-reporting-summary-flat.pdf

Life sciences study design

All studies must disclose on these points even when the disclosure is negative.

Sample size	No sample sizes to calculate.
Data exclusions	No exclusions
Replication	All experiments were performed in triplicate unless otherwise specified, which is the standard replicate size for the types of experiments

conducted for this manuscript. Graphs depict calculated SEM from all replicates. Statistical analyses were performed using Student's t-test or one-way ANOVA and represented as follows: *<0.05, **<0.01, ***<0.001, and ****<0.0001.

Randomization	Not applicable to this study
Blinding	For DNA-fiber length determination, fibers were imaged on a Nikon Eclipse Ni epi-fluorescent microscope using an oil-immersion 40x objective. Images were acquired using the NIS-Elements software. All samples were blinded when being imaged and analyzed. At least 100 fibers were scored per experiment, which were repeated in triplicate. The data shown were pooled from independent experiments

Reporting for specific materials, systems and methods

We require information from authors about some types of materials, experimental systems and methods used in many studies. Here, indicate whether each material, system or method listed is relevant to your study. If you are not sure if a list item applies to your research, read the appropriate section before selecting a response.

Materials & experimental systems

n/a	Involved in the study	
☐	☒	Antibodies
☐	☒	Eukaryotic cell lines
☐	☐	Palaeontology and archaeology
☐	☐	Animals and other organisms
☐	☐	Clinical data
☐	☐	Dual use research of concern
☐	☐	Plants

Methods

n/a	Involved in the study	
☒	☐	ChIP-seq
☐	☒	Flow cytometry
☒	☐	MRI-based neuroimaging

Antibodies

Antibodies used

Flag M2 (mouse monoclonal) Sigma A8592-.2MG
PP2A Aβ subunit (goat polyclonal) Santa Cruz sc-6113
PP2A B subunit (100C1) (rabbit monoclonal) CST 2290S
PP2A C subunit (1D6) (mouse monoclonal) Santa Cruz sc-80665
c-Myc (9E10) (mouse monoclonal) Santa Cruz sc-40
p107 (C-18) (rabbit polyclonal) Santa Cruz sc-318
pRB (G3-245) (mouse monoclonal) BD 554136
RB2 (Clone 10) (mouse monoclonal) BD 610262
Cyclin A2 (E6D1J) XP (rabbit monoclonal) CST 67955S
Cyclin E1 (D7T3U) (rabbit monoclonal) CST 20808
Cyclin D1 Antibody (A-12) (mouse monoclonal) Santa Cruz sc-8396
CDK2 (E8J9T) XP (rabbit monoclonal) CST 18048S
GAPDH (0411) (mouse monoclonal) Santa Cruz sc-47724
GAPDH (1E6D9) (mouse monoclonal) Proteintech 60004-1-Ig
Phospho-Akt (Ser473) (D9E) XP (rabbit monoclonal) CST 4060S
Phospho-Akt (Thr308) (244F9) (rabbit monoclonal) CST 4056S
Akt Antibody (rabbit polyclonal) CST 9272S
Phospho-GSK-3α/β (Ser21/9) (D17D2) (rabbit monoclonal) CST 8566S
GSK-3β (D5C5Z) XP (rabbit monoclonal) CST 12456S
Phospho-p44/42 MAPK (Erk1/2) (Thr202/Tyr204) (rabbit polyclonal) CST 9101S
ERK 1 (K-23) (rabbit polyclonal) Santa Cruz sc-94
FAM122A (3E9) (mouse monoclonal) Abnova H00116224-M03
Phospho-cdc2 (Tyr15) (rabbit monoclonal) CST 9111S
cdc2 (POH1) (mouse monoclonal) CST 9116S
β-Actin (C4) (mouse monoclonal) Santa Cruz sc-47778
Phospho-Chk2 (Thr68) (C13C1) (rabbit monoclonal) CST 2197S
Chk2 (rabbit polyclonal) CST 2662S
Phospho-Chk1 (Ser345) (133D3) (rabbit monoclonal) CST 2348S
Chk1 (2G1D5) (mouse monoclonal) CST 2360S
p21 Waf1/Cip1 (12D1) Rabbit mAb CST 2947
p53 OP-43 mouse monoclonal Calbiochem DO-1
BrdU (bu20a) (mouse monoclonal) CST 5292S
anti-BrdU (IdU) (mouse monoclonal) BD Biosciences 347580
anti-BrdU (CldU) (rat monoclonal) Abcam ab6326
Anti-FAM122A (rabbit polyclonal) Sigma/Atlas Antibodies HPA056646-100UL
PP2A-B55-alpha (mouse monoclonal) Santa Cruz sc-81606
Phospho-Histone H2A.X (Ser139) (D7T2V) (mouse monoclonal) CST 80312S
Alexa Fluor 488 Goat Anti-Rabbit Invitrogen A11034

	Alexa Fluor 594 Goat Anti-Mouse Invitrogen A11032<br>ECL Rabbit IgG, HRP-linked whole Ab (from donkey) GE Healthcare NA934V<br>ECL Mouse IgG, HRP-linked whole Ab (from donkey) GE Healthcare NA931V<br>Mouse anti-goat IgG-HRP Santa Cruz sc-2354<br>Monoclonal ANTI-FLAG M2 antibody produced in mouse, ANTI-FLAG M2 Affinity Agarose Gel Sigma A2220<br>Anti-c-Myc Agarose Affinity Gel antibody produced in rabbit (polyclonal) Sigma A7470<br>RFP-Trap agarose beads Chromotek rta-20<br>GFP-Trap agarose beads Chromotek gta-10
Validation	These are all commercial antibodies that were selected based on previous validation in the literature. In addition, antibodies to FAM122A (3E9, mouse monoclonal, Abnova H00116224-M03) and PP2A B subunit (100C1, rabbit monoclonal, CST 2290S) have been validated in our lab using knock out cell lines.

Eukaryotic cell lines

Policy information about cell lines and Sex and Gender in Research

Cell line source(s)	All cell lines were obtained from ATCC or from authenticated stocks of the Fox Chase Cancer Center (FCCC) Cell Culture facility.
Authentication	Cell lines used were from early stocks from cells received from ATCC or authenticated at the FCCC cell culture facility.
Mycoplasma contamination	All cell lines were tested for mycoplasma biannually or if there were any signs of stress.
Commonly misidentified lines<br>(See ICLAC register)	None

Palaeontology and Archaeology

Specimen provenance	*Provide provenance information for specimens and describe permits that were obtained for the work (including the name of the issuing authority, the date of issue, and any identifying information). Permits should encompass collection and, where applicable, export.*
Specimen deposition	*Indicate where the specimens have been deposited to permit free access by other researchers.*
Dating methods	*If new dates are provided, describe how they were obtained (e.g. collection, storage, sample pretreatment and measurement), where they were obtained (i.e. lab name), the calibration program and the protocol for quality assurance OR state that no new dates are provided.*

☐ Tick this box to confirm that the raw and calibrated dates are available in the paper or in Supplementary Information.

Ethics oversight	*Identify the organization(s) that approved or provided guidance on the study protocol, OR state that no ethical approval or guidance was required and explain why not.*

Note that full information on the approval of the study protocol must also be provided in the manuscript.

Animals and other research organisms

Policy information about studies involving animals; ARRIVE guidelines recommended for reporting animal research, and Sex and Gender in Research

Laboratory animals	*For laboratory animals, report species, strain and age OR state that the study did not involve laboratory animals.*
Wild animals	*Provide details on animals observed in or captured in the field; report species and age where possible. Describe how animals were caught and transported and what happened to captive animals after the study (if killed, explain why and describe method; if released, say where and when) OR state that the study did not involve wild animals.*
Reporting on sex	*Indicate if findings apply to only one sex; describe whether sex was considered in study design, methods used for assigning sex. Provide data disaggregated for sex where this information has been collected in the source data as appropriate; provide overall numbers in this Reporting Summary. Please state if this information has not been collected. Report sex-based analyses where performed, justify reasons for lack of sex-based analysis.*
Field-collected samples	*For laboratory work with field-collected samples, describe all relevant parameters such as housing, maintenance, temperature, photoperiod and end-of-experiment protocol OR state that the study did not involve samples collected from the field.*
Ethics oversight	*Identify the organization(s) that approved or provided guidance on the study protocol, OR state that no ethical approval or guidance was required and explain why not.*

Note that full information on the approval of the study protocol must also be provided in the manuscript.

Clinical data

Policy information about clinical studies

All manuscripts should comply with the ICMJE guidelines for publication of clinical research and a completed CONSORT checklist must be included with all submissions.

Clinical trial registration	*Provide the trial registration number from ClinicalTrials.gov or an equivalent agency.*
Study protocol	*Note where the full trial protocol can be accessed OR if not available, explain why.*
Data collection	*Describe the settings and locales of data collection, noting the time periods of recruitment and data collection.*
Outcomes	*Describe how you pre-defined primary and secondary outcome measures and how you assessed these measures.*

Dual use research of concern

Policy information about dual use research of concern

Hazards

Could the accidental, deliberate or reckless misuse of agents or technologies generated in the work, or the application of information presented in the manuscript, pose a threat to:

No	Yes	
☒	☐	Public health
☒	☐	National security
☒	☐	Crops and/or livestock
☒	☐	Ecosystems
☒	☐	Any other significant area

Experiments of concern

Does the work involve any of these experiments of concern:

No	Yes	
☒	☐	Demonstrate how to render a vaccine ineffective
☒	☐	Confer resistance to therapeutically useful antibiotics or antiviral agents
☒	☐	Enhance the virulence of a pathogen or render a nonpathogen virulent
☒	☐	Increase transmissibility of a pathogen
☒	☐	Alter the host range of a pathogen
☒	☐	Enable evasion of diagnostic/detection modalities
☒	☐	Enable the weaponization of a biological agent or toxin
☒	☐	Any other potentially harmful combination of experiments and agents

Plants

Seed stocks	*Report on the source of all seed stocks or other plant material used. If applicable, state the seed stock centre and catalogue number. If plant specimens were collected from the field, describe the collection location, date and sampling procedures.*
Novel plant genotypes	*Describe the methods by which all novel plant genotypes were produced. This includes those generated by transgenic approaches, gene editing, chemical/radiation-based mutagenesis and hybridization. For transgenic lines, describe the transformation method, the number of independent lines analyzed and the generation upon which experiments were performed. For gene-edited lines, describe the editor used, the endogenous sequence targeted for editing, the targeting guide RNA sequence (if applicable) and how the editor was applied.*
Authentication	*Describe any authentication procedures for each seed stock used or novel genotype generated. Describe any experiments used to assess the effect of a mutation and, where applicable, how potential secondary effects (e.g. second site T-DNA insertions, mosiacism, off-target gene editing) were examined.*

Flow Cytometry

Plots

Confirm that:

- [x] The axis labels state the marker and fluorochrome used (e.g. CD4-FITC).
- [x] The axis scales are clearly visible. Include numbers along axes only for bottom left plot of group (a 'group' is an analysis of identical markers).
- [x] All plots are contour plots with outliers or pseudocolor plots.
- [x] A numerical value for number of cells or percentage (with statistics) is provided.

Methodology

Sample preparation

Collection for flow cytometric DNA content analysis was performed by collecting the cells followed by ice-cold ethanol fixation. Following 2 PBS washes, cells were stained with 1x propidium iodide solution (in 1% FBS in PBS with 1mg/mL RNase A) in the dark for 30 minutes.
For cells expressing mGFP (TagGFP), cells were fixed in ice-cold methanol to preserve GFP fluorescence and processed as samples fixed with 70% ethanol.

BrdU incorporation assays in HEK293 cells were performed by the addition of 10µM BrdU to cells for a 45-minute pulse. Cells were fixed using ice-cold 70% ethanol and left overnight for permeabilization. Cells were washed with PBS twice followed by denaturing with 2N HCl for 20 min. Following washes and neutralization with 0.1M Na2B4O7 for 10 min, cells were incubated with anti-BrdU antibody (1:200) for 20 min. Subsequent washes were followed up by anti-rabbit Alexa Fluor 488 secondary (1:200) for 10 min. Following two additional washes, cells were stained with 1x propidium iodide solution as described above.

EdU incorporation assays were performed using the APEXBio EdU Flow Cytometry Assay Kits (Cy5) (K1078). Briefly, a 10 µM final concentration of EdU was added to cells 1 h prior to collection and the cells washed and fixed in 4% PFA in PBS. Cells were permeabilized using a 1x saponin-based permeabilization agent (PBS pH 7.4), 1% BSA, 0.1% NaN3, and 0.1% saponin) and subject to the Cy5 azide click-chemistry reaction for 30 min. Following additional wash steps with the saponin-permeabilization reagent, cells were stained with 1x propidium iodide solution.

Instrument

BD LSR-II

Software

Analyzed in FlowJo v10 (BD Biosciences)

Cell population abundance

We did not sort.

Gating strategy

Gating based on PI and/or BrdU/EdU intensity. Secondary antibody with no primary antibody stained cells were used as negative control for gating the negative population.

- [x] Tick this box to confirm that a figure exemplifying the gating strategy is provided in the Supplementary Information.
